# Towards quality adolescent-friendly services in TB care

**DOI:** 10.5588/ijtld.21.0059

**Published:** 2021-07-01

**Authors:** K. M. Laycock, J. Eby, T. Arscott-Mills, S. Argabright, C. Caiphus, B. Kgwaadira, E. D. Lowenthal, A. P. Steenhoff, L. A. Enane

**Affiliations:** 1 Division of Infectious Diseases, Department of Pediatrics, The Children’s Hospital of Philadelphia, Philadelphia, PA; 2 Department of Pediatrics, Boston Children’s Hospital, and Department of Medicine, Brigham and Women’s Hospital, Boston, MA; 3 Department of Pediatrics, The Children’s Hospital of Philadelphia, Philadelphia, PA; 4 University of Pennsylvania Perelman School of Medicine, Philadelphia, PA, USA; 5 Botswana-UPenn Partnership, Gaborone, Botswana; 6 University of Pennsylvania, Philadelphia, PA, USA; 7 Botswana National TB Programme, Ministry of Health, Gaborone, Botswana; 8 The Ryan White Center for Pediatric Infectious Disease and Global Health, Department of Pediatrics, Indiana University School of Medicine, Indianapolis, IN, USA

Dear Editor,

Each year, 1.8 million adolescents and young adults (AYA, ages 10–24 years) develop TB.^1^,^2^ This number was only recently estimated, as routine TB surveillance strategies traditionally divide cases among “children” (ages 0–14) and “adults” (ages ≥15), neglecting adolescents.^1^,^2^ AYA, particularly those with TB-HIV coinfection, bear increased risk for adverse outcomes compared to older adults.^3^–^6^ Nevertheless, AYA remain neglected in TB services, research, and policy.^1^,^7^ AYA have unique health needs requiring dedicated approaches.^8^ This manifests particularly when engaging in care for highly stigmatized and complex diseases, such as TB and HIV.^7^,^9^–^12^ The WHO has issued guidance for the provision of quality adolescent-friendly services (AFS).^13^ These standards outline dimensions of equitable, accessible, acceptable, appropriate, and effective health services for AYA. Youth-centered approaches are increasingly implemented and studied in a range of health services, including for HIV.^14^,^15^ However, TB services have not been tailored to AYA needs.^1^,^7^

We conducted a qualitative study of healthcare workers (HCWs) perspectives on needed adaptations for adolescent-friendly TB care. In-depth interviews exploring specific challenges and approaches in the care of AYA with TB were conducted with 16 HCWs at nine public primary care clinics in Gaborone, Botswana, between June and August 2016, as previously described.^7^ All HCWs provided informed consent and the protocol was approved by the relevant ethical review boards.^7^ In this analysis, we examined HCW perspectives on adolescent-friendly TB care using the WHO framework.^13^ A codebook was established with the dimensions and characteristics of AFS, with their definitions.^13^ After establishing inter-rater reliability and consensus, two team members (LAE and KML) independently coded transcripts using Dedoose v8.3 (Sociocultural Research Consultants, Los Angeles, CA, USA). The team discussed findings and reached consensus around emerging themes. These are summarized here, with illustrative excerpts.^13^ The HCWs identified areas of intervention in all dimensions of AFS and discussed approaches to optimally support AYA with TB and TB-HIV infection through quality health services (Table).

**Table:** Emergent themes from in-depth interviews with HCWs on unmet needs and areas of intervention for quality adolescent-friendly services for AYA with TB or TB-HIV infection ^*^

Dimensions of adolescent-friendly services^13^	Themes in AYA TB and TB-HIV care	Example excerpts
Equitable: All AYA are able to obtain health services regardless of status	Younger adolescents require parental consent Non-citizens unable to access ART at the time of interviews^†^ HCWs aimed to provide “friendly” care Unmet needs of vulnerable AYA	“We established a very good rapport... I wanted her to feel more comfortable, because of the [HIV] status. ... I tried to close the gap, so she doesn’t feel that if you have this status it’s like you are not like others.” “Some [AYA] come from dysfunctional families. ... Some of them they don’t stay with their parents, they stay with their siblings, who work long hours. ... Most of the time those that are coming from dysfunctional families [including situations of abuse and neglect] and those that are orphans, you encounter such problems [with adherence to treatment].” I think [AYA on community-based DOT are] doing well because most of...these adolescents, they’re students, some of them they’re going to school, they’re working. So, you know, *gore* [‘because’] you can’t just miss school every day, you’re always late, because you have to go to the facility to take your treatment. So it’s best when you put them on community TB care. They do well because...*akare* [‘isn’t it’]... some of them they understand, they understand, they know about TB. Their parents have taken treatment so they adhere to treatment, they know. So when you’re giving them community TB [treatment], they take. “I think it depends on where they [adolescents] come from. Like, when they have support, we never experience any problem, even with the collection of sputum, even with the completion [of TB treatment], even with the HIV test, but I imagine what can happen if there’s no support from home. But if there’s support, you always see the mother or the very person who is nearer to the patient bringing the patient every time for the supply, for the check-ups, that’s the support I mean.” “My experience is: young patients, they are difficult to manage. Most of them they don’t come for follow-ups. Most of them they are afraid of stigma and discrimination. Most of them they are schooling, so in this case of schooling and taking the medication, it’s difficult on their schedule. They can’t adhere to the medications. They can’t do their follow-ups. So... I don’t know what to say more about them, but what I know is that they’re afraid of stigma and discrimination from their peers.” “We have community nurses they usually do the home visits to their clients. Yeah, they take the medication there. They do a follow-up. They give the injectables at home. They give medications at home. They came with the transport, like almost every day, once in a week, or twice in a week, they do so. But, the problem we had for now, we have a shortage of manpower and transport for now. Most of the time, we have an issue of transport. I cannot work...walk long distance visits...do the visits with no transport. No, I cannot do that. So, most of the time, they are being supported by the community health nurses.” “The first thing we can do: our waiting time, for them, there should always be a nurse that is available. To assist them.” “They can just come and meet as adolescents who are taking TB treatment, just them alone there and they share their experiences, challenges, ...and advise others who are finding it hard to live with the fact that they’re taking the treatment or they’re infected with TB. ...A peer group.” “I think [there is TB stigma], but we try to teach our patients that everyone is at risk for TB. I think when they leave this room, or after they complete treatment, they are different people unlike when they came, with the knowledge they’ve acquired about TB, and I think they can be the best teachers for others.” “Some [AYA] ended up searching me on WhatsApp. They found me on WhatsApp. That is when they started to communicate with me, not face-to-face.” “They know a lot. They hear a lot. They search a lot. Because some of them, they come with this reading information they have from the Internet or from their friends. But adults (laughs)... they just agree with everything that the HCW say (laughs). With the adolescents, they read a lot. They can even know much more than you yourself will know as a HCW. They know a lot.” “Those that are having TB and HIV... they have a really low self-esteem. ... You know some of them they’re just so hopeless. You see, because of this HIV they [think], “I can never get better” even if they take their medication – that’s why you can find problems that some of they can even miss.” “We have different approach and skills. ... We don’t work alone; we have social workers; we have psychologists. ... Normally, [if] I can’t manage that person, I can refer. They have that skill to approach someone. ... So usually, I refer so that they can tackle those situations.” “Honestly when, for example, if the patient, the child herself, let’s say it’s an orphan, we’d refer them to social workers. Even for counseling, we refer them to the social workers. We don’t really do that much intensive counseling in the facility, we just refer them to the social workers for any support that they may need emotionally, socially, physically, financially, because most of them if they’re orphans, they’ll need the system of a social worker.” “We try to be more friendly, as if you are doing the youth-friendly services. Of course, we don’t have here, but we try to, when we are dealing with [AYA], to use aspects of youth-friendly services so that they become more open and more relaxed.” “Maybe if there was time and transport to visit them at their home to show, really, to show them that you support them. It’s not like it’s the end of the world for them. And when some of these ones, when they see you coming to their home to educate and support them, that’s something to them. Even the treatment, if they’re telling you *gore* [‘because’] I can’t come in the morning to take treatment, DOT, maybe every morning if there was transport, you go and give them the supply.”
Accessible: AYA are able to obtain the services that are available Characteristics include: Free or affordable care Convenient hours Information about reproductive health services and how to access them Community support for AYA to obtain services Some services are provided by community members, outreach workers, and AYA	Conflicts with school or work Convenient hours provided where feasible Challenges for mobile AYA to access care at different sites Information provided for HIV and STI testing Pervasive stigma is a central barrier to care for AYA Need for social and family support for AYA in TB care Community-based DOT supports care access for AYA Interest in peer support models for TB care	
Acceptable: Adolescents are willing to obtain the available services Characteristics include: Confidentiality Privacy Non-judgmental, friendly providers Timeliness of care Care with or without an appointment Appealing care environments Information and education through varied channels AYA involved in designing, assessing, providing health services	Need for short wait times for AYA Interest in dedicated clinic times or spaces for AYA HCWs provide counseling for education and treatment adherence Can increase awareness and education about TB on social media or using mobile applications Interest in peer support models for TB care	
Appropriate: The needed services are provided, at the point of service or through referral linkages	Access to HIV care and support Referral may be needed for psychosocial support Need for programs to manage alcohol or substance abuse Needed supports for AYA experiencing food or financial insecurity	
Effective: The right health services are provided in the right way and make a positive contribution to health Characteristics include: Provider competencies to work with AYA Evidence-based protocols and guidelines Sufficient time to work effectively with AYA Availability of needed equipment, supplies, basic services to deliver care	Need for HCWs with dedicated training to provide care to AYA Lack of guidelines for managing AYA with TB Need for adequate staffing and material resources to meet AYA needs, e.g. through intensive counseling and home visits	

^*^ Semi-structured interviews were conducted at nine high-volume TB clinics with HCWs who regularly treated TB patients and had experience caring for AYA with TB. Two trained researchers (LAE and JE) conducted the interviews in English between June and August 2016. Responses were audio-recorded and transcribed. Transcripts did not contain identifying information. Minimal demographic data were recorded to protect the identity of participants. Recruitment of HCWs continued until transcripts reached thematic saturation surrounding the research questions.

^†^ This restriction was in place at the time of interviews. ART is now offered for free to non-citizens in Botswana.

AYA = adolescent and young adult; ART =antiretroviral therapy; HCW = healthcare worker; STI = sexually transmitted infection; DOT = directly observed therapy.

“Equitable care” ensures that all AYA can obtain available health services, regardless of status.^13^ Few restrictions on care were described. Non-citizen AYA could not access HIV treatment at the time of the interviews. HCW described unmet needs among vulnerable AYA that could limit access and completion of TB treatment, e.g., for those with limited family support or food insecurity. HCW discussed available supports for vulnerable AYA, such as social work referral. Otherwise, HCWs strove to provide “friendly” care to AYA and build rapport with them to ensure successful treatment.

“Accessible care” describes the provision of free or affordable health services, convenient hours, community support for AYA to access care, and provision of some health services by community members, outreach workers, or AYA themselves.^13^ HCWs viewed social support as key to AYA engagement in TB and HIV treatment. They described anticipated stigma as a barrier to care access and a central cause of loss to follow-up among AYA. Some HCWs suggested expanding awareness efforts in the community to address TB and HIV stigma. HCWs further noted that strengthening community-based TB care models could facilitate access to services, allowing AYA to avoid missing school or work.

“Acceptable care” refers to the willingness of AYA to obtain available health services.^13^ Elements of acceptable care include confidentiality, privacy, nonjudgmental HCWs, short waiting times, services with or without an appointment, appealing care environments, health education through a variety of channels, and active involvement of adolescents in designing, assessing, and providing health services.^13^ HCWs noted multiple factors impacting acceptability of TB services to AYA. Stigma influences both access to care and acceptability of available services, with long waiting times and AYA concerns about privacy and confidentiality while accessing TB care presenting an important barrier. HCWs also described that AYA were reluctant to miss school or work obligations to access services. To improve acceptability of health services, HCWs suggested establishing adolescent-dedicated clinic times or spaces. HCWs further suggested forming peer support groups for AYA with TB or TB-HIV. They described observing positive impacts of peer support for HIV, and nearly every participant suggested that peer support models for TB care may be impactful. Finally, HCWs pointed to opportunities to increase AYA health education through internet and social media sources, and to support care engagement through mHealth tools, such as through texting apps used by AYA.

“Appropriate care” describes the provision, either directly or through referral, of all necessary health services.^13^ HCWs noted that while they provided counseling to AYA, some AYA needed greater psychological support. They also discussed impacts of alcohol use for some AYA with TB – including for their general health and risk for hepatotoxicity while taking TB treatment – and a lack of dedicated programs for managing alcohol dependence.

“Effective care” encompasses the availability of trained providers to work with adolescents; sufficient time for HCWs to manage adolescents; evidence-based protocols and guidelines; and essential equipment, supplies, and basic services to deliver health services.^13^ HCWs described not having specific training to work with AYA. Many commented on the lack of differentiation between TB services for AYA and for older adults. Like others globally, the Botswana National TB Programme lacks dedicated protocols for AYA. HCWs nevertheless recognized that AYA need dedicated approaches, including more intensive counseling. Inadequate staffing meant that HCWs lacked sufficient time to adequately address AYA concerns, in particular, the complex psychosocial needs of AYA with TB-HIV. HCWs noted that home visits provided opportunities for comprehensive assessment of AYA needs. However, inadequate staffing and lack of transportation limited these visits. Finally, stock-outs of TB medications posed a challenge. Identified areas of intervention therefore included: training and expertise in AFS; adequate HCW staffing to allow sufficient time for AYA, particularly for psychosocial complexities of TB-HIV; dedicated guidelines for AYA TB care; and resources for home visits.

Limitations of this study include that we were unable to include AYA perspectives, and that interview guides were not strictly framed around the WHO dimensions of AFS. Therefore, this is not an exhaustive exploration, but an initial step to consider areas of intervention to provide quality AFS to AYA with TB.

This study examines routine TB care within the dimensions of the WHO framework for quality AFS.^13^ HCWs emphasized opportunities to address isolation and stigma, and to facilitate greater AYA engagement in TB care. These included putting resources towards psychosocial counseling, home visits, and establishing peer support models. Further work is needed to examine perspectives on TB services among AYA themselves, particularly to ensure that services are equitable, accessible, and acceptable. AYA participation in the design and implementation of health services forms a critical element of adolescent-friendly care. The WHO framework for AFS provides a useful basis for tailoring TB services to AYA needs. Innovative strategies are needed to provide quality AFS to AYA with TB and TB-HIV, to address AYA needs and improve outcomes.

## References

[1] Snow KJ (2020). Adolescent tuberculosis. Lancet Child Adolesc Health.

[2] Snow KJ (2018). The incidence of tuberculosis among adolescents and young adults: a global estimate. Eur Respir J.

[3] Berry KM (2019). Treatment outcomes among children, adolescents, and adults on treatment for tuberculosis in two metropolitan municipalities in Gauteng Province, South Africa. BMC Public Health.

[4] Enane LA (2019). Investigating outcomes of adolescents and young adults (10–24 years of age) lost to follow-up from tuberculosis treatment in Gaborone, Botswana. Pediatr Infect Dis J.

[5] Enane LA (2016). Loss to follow-up among adolescents with tuberculosis in Gaborone, Botswana. Int J Tuberc Lung Dis.

[6] Snow K (2017). Tuberculosis in adolescents and young adults: epidemiology and treatment outcomes in the Western Cape. Int J Tuberc Lung Dis.

[7] Enane LA (2020). TB and TB-HIV care for adolescents and young adults. Int J Tuberc Lung Dis.

[8] Sawyer SM (2007). Adolescents with a chronic condition: challenges living, challenges treating. Lancet.

[9] Enane LA (2020). “A problem shared is half solved” - a qualitative assessment of barriers and facilitators to adolescent retention in HIV care in western Kenya. AIDS Care.

[10] Enane LA, Christenson JC (2021). Global emerging resistance in pediatric infections with TB, HIV, and gram-negative pathogens. Paediatr Int Child Health.

[11] Enane LA (2018). Traversing the cascade: urgent research priorities for implementing the ’treat all’ strategy for children and adolescents living with HIV in sub-Saharan Africa. J Virus Erad.

[12] Enane LA, Vreeman RC, Foster C (2018). Retention and adherence: global challenges for the long-term care of adolescents and young adults living with HIV. Curr Opin HIV AIDS.

[13] World Health Organization. (2012). Making health services adolescent friendly: developing national quality standards for adolescent friendly health services.

[14] Teasdale CA (2016). Impact of youth and adolescent friendly services on retention of 10–24-year-olds in HIV care and treatment programs in Nyanza, Kenya. J Acquir Immune Defic Syndr.

[15] Zanoni BC (2019). Barriers to retention in care are overcome by adolescent-friendly services for adolescents living with HIV in South Africa: a qualitative analysis. AIDS Behav.

